# *IL4* Gene Variants rs2243250 and rs2243248 and Their Association with Clinical Phenotypes of Severe Asthma in the Mexican Population: In Silico Functional Analysis and Regulatory Implications

**DOI:** 10.3390/ijms27114711

**Published:** 2026-05-23

**Authors:** Ingrid Berenice Montoya-Delgado, Itzel Vianey Ochoa-García, Zaira Lorena Escobedo-Salcedo, Margarita Ortega-Cisneros, Alicia Del Toro-Arreola, Adrián Daneri-Navarro, Yeminia Valle, María Enriqueta Nuñez-Nuñez, Beatriz Bayardo-Gutierrez, Antonio Quintero-Ramos

**Affiliations:** 1 Instituto de Investigación en Inmunología, Departamento de Fisiología, Centro Universitario de Ciencias de la Salud, Universidad de Guadalajara, Guadalajara 44340, Jalisco, Mexico; ingrid.montoya9883@alumnos.udg.mx (I.B.M.-D.); alicia.deltoro@academicos.udg.mx (A.D.T.-A.); adrian.daneri@academicos.udg.mx (A.D.-N.); 2 Doctorado en Genética Humana, Departamento de Biología Molecular y Genómica, Centro Universitario de Ciencias de la Salud, Universidad de Guadalajara, Guadalajara 44340, Jalisco, Mexico; 3 Departamento de Inmunología Clínica y Alergia, Hospital de Especialidades, Centro Médico Nacional de Occidente, Instituto Mexicano del Seguro Social, Guadalajara 44340, Jalisco, Mexico; ileochoa_1@hotmail.com (I.V.O.-G.); zairalore@hotmail.com (Z.L.E.-S.); maorcis@gmail.com (M.O.-C.); 4 Instituto de Neurociencias Traslacionales, Departamento de Neurología, Centro Universitario de Ciencias de la Salud, Universidad de Guadalajara, Guadalajara 44340, Jalisco, Mexico; yeminia.valle@academicos.udg.mx; 5 Servicio de Alergia e Inmunología Clínica Pediátrica, Hospital Civil de Guadalajara “Dr. Juan I. Menchaca”, Guadalajara 44340, Jalisco, Mexico; nunezen@hotmail.com (M.E.N.-N.); beabg24@gmail.com (B.B.-G.); 6 Unidad de Investigación Biomédica 02, Unidad Médica de Alta Especialidad, Hospital de Especialidades, Centro Médico Nacional de Occidente, Instituto Mexicano del Seguro Social, Guadalajara 44340, Jalisco, Mexico

**Keywords:** severe asthma, *IL4* gene, genetic variants, in silico analysis

## Abstract

Severe asthma (SA) is a chronic respiratory disease characterized by clinical heterogeneity and poor therapeutic response. Variants in the *IL4* gene, including rs2243250 and rs2243248, have been associated with asthma susceptibility and severity in different populations; however, their role in the Mexican population remains unclear. This study evaluated the association of *IL4* promoter variants rs2243250 and rs2243248 with SA and related clinical characteristics in a Mexican population using genetic and in silico approaches. In total, 106 patients with SA and 180 healthy individuals were included. Genotyping was performed using allelic discrimination assays with TaqMan^®^ probes, and associations between genotypes and clinical variables were assessed. No significant differences in allele or genotype frequencies were observed between groups. However, the rs2243250 TT genotype was associated with nocturnal symptoms (OR = 3.03, 95% CI = 1.31–7.00, *p* = 0.009) and increased use of rescue medication (OR = 3.16, 95% CI = 1.41–7.07, *p* = 0.005). The rs2243248 TG/GG genotypes were associated with epithelial allergy (*p* < 0.05). In silico analysis suggested a regulatory role for both variants. These findings suggest that *IL4* variants may not influence overall disease risk but could modulate clinical features of asthma severity.

## 1. Introduction

Chronic respiratory diseases, such as asthma, are among the leading causes of morbidity and mortality worldwide and contribute substantially to disability and reduced quality of life [1]. Asthma is a chronic inflammatory airway disease characterized by marked clinical and etiological heterogeneity, posing significant challenges for both diagnosis and management [2]. The classification of asthma severity is mainly based on clinical and functional parameters, as well as the intensity and dosage of treatment required. Severe asthma (SA), although less prevalent, affecting approximately 3 to 10 percent of the global population, represents a significant health problem due to its frequent exacerbations, the need for high-dose multiple medications, and resistance to standard therapies [3,4].

Type 2 inflammation, mediated by cytokines such as IL-4, IL-5 and IL-13 and characterized by eosinophilia, elevated Immunoglobulin E (IgE) levels, airway hyperresponsiveness and structural remodeling, plays a central role in the pathophysiology of allergic asthma [5]. Several genes involved in this pathway have been implicated in asthma susceptibility and severity, although findings vary across populations. Susceptibility loci have been identified at chromosome regions 2q12 (*IL1R* encoding the IL-33 receptor), 5q22 with *TSLP*, 9p24 containing *IL33* and 10p14 with *GATA3*, a key transcription factor for Th2 differentiation [6]. Other genetic studies have highlighted chromosomal regions associated with the development of SA, including 5q31-33, which harbors the *IL4* gene [7]. Single-Nucleotide Variants (SNVs) in the *IL4* gene, located within its regulatory region, including rs2243250 (−590 C>T) and rs2243248 (−1098 T>G), have attracted considerable attention due to their potential role in modulating IL-4 expression and consequently promoting Th2-driven inflammation. These variants are positioned within the promoter region, where they may influence transcriptional activity by altering the binding affinity of transcription factors such as NFAT. In particular, it has been suggested that the SNV rs2243250 increases promoter activity, which could lead to increased IL-4 expression [8]. Elevated IL-4 levels promote Th2 polarization, stimulate B-cell class switching to IgE, and enhance eosinophilic inflammation, all of which are key mechanisms in the pathophysiology of asthma. Therefore, the presence of the T allele may contribute to asthma susceptibility by amplifying these immune responses [9]. Additionally, reported allele frequencies and effect sizes vary across populations, with some studies showing a significant association between the T allele or TT genotype and increased asthma risk, while others report no association, highlighting the heterogeneity of genetic effects across different ethnic groups [10,11]. These contrasting findings emphasize the importance of population specific-analyses when evaluating genetic contributions to asthma and atopy [11,12].

SA is a complex disease influenced by both genetic and environmental factors, and its heterogeneous nature contributes to variability in clinical manifestations and therapeutic response [13]. Studying genetic variants that could contribute to these differences helps us better understand the biological basis of the disease. In addition, bioinformatics analyses can provide clues about the possible regulatory functions of these variants, complementing the genetic findings.

To date, no studies in Mexico have evaluated the association of *IL4* promoter SNVs rs2243250 and rs2243248 with this condition. Therefore, this study assessed their frequency in Mexican patients with SA and healthy subjects from the reference group and examined their relationship with clinical features. In addition, an in silico analysis was performed to predict the potential regulatory effects of these variants.

## 2. Results

### 2.1. Sociodemographic and Clinical Description

The demographic characteristics of both groups are summarized in Table 1. The mean age was significantly higher in patients with SA (51 years) compared with the RG (40 years). The proportion of women was also higher in the SA group (77%) than in the RG (54%). Body mass index (BMI) was greater in patients with SA (30.57 kg/m^2^) compared with the RG (26.5 kg/m^2^). No significant differences were observed in tobacco use between the two groups.

The most frequent treatment among SA patients was ICS–LABA, reported in 66% of cases, followed by oral corticosteroids in 42% and biologics in 41%. The most common comorbidities were allergic rhinitis, affecting 68% of patients, and chronic rhinosinusitis with nasal polyps, present in 45% (Table 2). Regarding clinical manifestations, half of the patients reported frequent nighttime symptoms, with a prevalence of 64%, while 53% had uncontrolled asthma. The most common sensitization was to environmental inhalants, observed in 80% of individuals (Table 3). Pulmonary function showed a mean FEV1 of 70.3%, FVC of 89.6%, and an FEV1/FVC ratio of 65%, while the mean serum IgE level was 293.7 IU/mL (Table 3).

### 2.2. Genotype Analysis of rs2243250 and rs2243248 Variants of IL4

The observed genotype frequencies in the reference group did not deviate significantly from Hardy–Weinberg equilibrium (HWE): rs2243248, *p* = 0.69; and rs2243250, *p* = 0.65.

For rs2243250, genotype frequencies were similar between patients with SA and RG. In the codominant model, neither the CT genotype (OR = 0.81; 95% CI: 0.46–1.43; *p* = 0.47) nor the TT genotype (OR = 0.57; 95% CI: 0.28–1.18; *p* = 0.15) showed significant associations. Likewise, no differences were observed under the dominant model (OR = 0.74; 95% CI: 0.43–1.27; *p* = 0.27) or the recessive model (OR = 0.65; 95% CI: 0.35–1.22; *p* = 0.22). At the allelic level, no significant association was observed for the T allele (OR = 0.77; 95% CI: 0.54–1.11; *p* = 0.16) (Table 4).

For rs2243248, genotype frequencies were also comparable between SA patients and the RG. In the codominant model, the TG genotype was not significantly associated with SA compared to the TT reference genotype (OR = 1.03; 95% CI: 0.53–1.95; *p* = 1). Due to the very low frequency of the GG genotype, reliable estimates could not be calculated. No differences were detected in the dominant model (OR = 1; 95% CI: 0.52–1.89; *p* = 0.99). Similarly, the recessive model could not be reliably evaluated due to the absence of GG homozygotes in the SA group. At the allelic level, no significant association was observed for the G allele (OR = 0.97; 95% CI: 0.55–1.70; *p* = 0.99) (Table 4). Similarly, additional genetic inheritance models, including codominant, dominant, recessive, over dominant, and log-additive models, were explored to further evaluate the association between the analyzed single-nucleotide variants and SA susceptibility. However, no statistically significant associations were identified under these additional models. Therefore, these exploratory analyses were not included.

In the SA group, the TT genotype of the rs2243250 variant was associated with the presence of nighttime symptoms (OR = 3.03; 95% CI = 1.31–7.00; *p* = 0.009, *p*FDR = 0.020) and with increased use of rescue medication (OR = 3.16; 95% CI = 1.41–7.07; *p* = 0.005, *p*FDR = 0.019). In contrast, the same TT genotype was associated with a lower likelihood of using combined ICS + LABA (budesonide) therapy (OR = 0.15; 95% CI = 0.03–0.76; *p* = 0.023, *p*FDR = 0.030). Similarly, the dominant allele of the rs2243248 variant (TG + GG vs. TT) showed an association with epithelial allergy (OR = 3.96; 95% CI = 1.07–14.6; *p* = 0.039, *p*FDR = 0.039) (Table 5). All reported associations remained statistically significant after false discovery rate (FDR) correction for multiple comparisons. Multivariate logistic regression models adjusted for age, sex, and BMI yielded similar effect estimates, and no relevant changes in the magnitude or statistical significance of the associations were observed.

### 2.3. Linkage Disequilibrium and Haplotypes

Comparisons between the groups showed no statistically significant differences in haplotype distribution (Table 6). Non-significant trends were observed: the CT haplotype was more frequent in cases, while the TT haplotype was more common in the reference group, but neither reached statistical significance. The linkage disequilibrium analysis of rs2243250 and rs2243248 revealed a high D′ value: 0.923, (*p* < 0.05), but a low r^2^ value: 0.086, indicating strong allele co-segregation without high allelic correlation.

### 2.4. In Silico Analysis of the Study Variants

The variants rs2243250 and rs2243248 were classified with rank 1f and a score of 0.55436 in RegulomeDB, indicating strong evidence of a regulatory role (Table 7). For rs2243250, binding sites for ZFX and ARID1B were identified, along with chromatin marks of active enhancers and accessibility signals in immune tissues and lung. In contrast, rs2243248 showed binding of ZFX and IKZF1, together with evidence of active enhancers and chromatin accessibility in immune cells and the T47D cell line.

HaploReg analysis showed that rs2243250 is associated with changes in Sox and MAX motifs, while rs2243248 alters an Osr binding motif. These findings complement the experimental evidence from RegulomeDB, suggesting that both variants exhibit regulatory activity, although rs2243250 displays a broader spectrum of transcription factor interactions.

**Figure 1:**
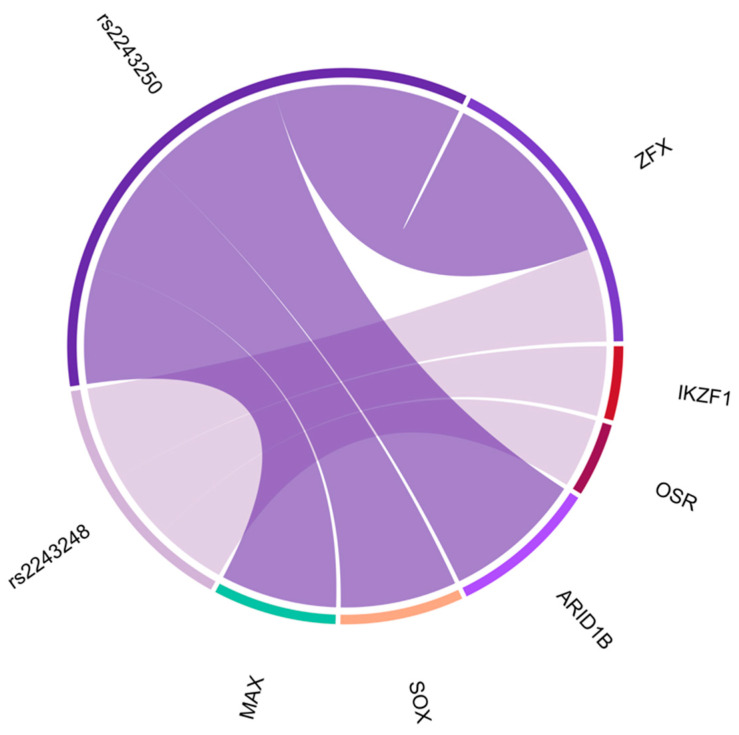
Predicted interactions between *IL4* promoter variants and transcription factors (TFs). The rs2243250 variant showed interactions with ZFX, ARID1B, Sox, and MAX family members, whereas rs2243248 was associated with ZFX, IKZF1, and Osr. These transcription factors are involved in regulatory pathways related to immune response, chromatin accessibility, and transcriptional regulation, supporting the potential functional relevance of these variants in asthma. This figure was generated using R version 4.3.1.

Figure 1 graphically represents the interactions between the SNVs and transcription factors (TFs). In this analysis, rs2243250 was associated with ZFX, ARID1B, Sox, and MAX family members, whereas rs2243248 was linked to ZFX, IKZF1, and Osr. These findings suggest that rs2243250 exhibits a more complex regulatory profile compared to rs2243248.

### 2.5. Sensitivity Power Analysis

For the case–reference group comparison (106 SA patients vs. 180 reference group; α = 0.05, two-sided, log-additive model), the study achieved approximately 80% power to detect minimum allelic odds ratios (ORs) of 1.63 for rs2243250 (minor allele frequency [MAF] ≈ 0.49) and 1.98 for rs2243248 (MAF ≈ 0.11). Statistical power increased to 92% for an OR of 1.8 in rs2243250 and 81% for an OR of 2.0 in rs2243248. For subgroup analyses within the SA cohort, statistical sensitivity was lower due to reduced sample size within genotype strata, with approximately 80% power limited to detecting ORs of ~6.0 or greater [14].

## 3. Discussion

In recent decades, the prevalence of allergic diseases, particularly asthma, has shown a steady increase and currently represents the most common chronic respiratory disease worldwide, affecting hundreds of millions of people and constituting a major public health concern both in Mexico and globally [1]. Several recent studies have demonstrated that specific genetic variants play an important role in asthma susceptibility. To date, numerous genes have been identified in association with this disease, including *STAT6*, *ORMDL3*, *GSDMB*, *IL1RL1*, *IL18R1*, *IL4*, *IL13*, and *IL2RA*, all of which participate in key immunological processes related to inflammation and allergic responses [15,16,17].

In this work, we evaluated the contribution of two promoter SNVs, rs2243250 (−590 C>T) and rs2243248 (−1098 T>G), of the *IL4* gene in a Mexican population with SA, integrating genetic association analyses with clinical phenotyping and in silico functional predictions. This integrative approach allows not only the assessment of disease susceptibility but also the exploration of how genetic variation supports specific clinical manifestations of asthma. We observed that patients with SA had a higher mean age and a predominance of females, findings consistent with previous reports in both Latin American and global populations [18,19,20,21]. The higher prevalence of SA among women has been associated with hormonal influences, particularly the modulatory effects of estrogens and progesterone on type 2 immune responses. These hormones enhance Th2-driven inflammation and promote cytokine production, including IL-4, whereas androgens may exert a protective immunomodulatory effect [20,21,22]. This sex-related immunological bias is particularly relevant in the context of *IL4* genetic variation, as increased IL-4 activity may amplify type 2 inflammatory pathways and contribute to disease severity and poor clinical control.

Despite this, no significant association was observed between the evaluated *IL4* promoter SNVs and overall susceptibility to SA, this finding is consistent with the heterogeneous results reported across different populations, where the contribution of these variants appears to be modest and highly context-dependent [7,23,24]. Several factors may explain the lack of association, including population-specific genetic backgrounds, differences in allele frequencies, gene–environment interactions, and the complex polygenic nature of asthma, in which individual variants often exert small effects [25,26,27]. Significant associations were identified between these SNVs and specific clinical features. In particular, under the recessive model, the TT genotype of rs2243250 was associated with an increased risk of nocturnal symptoms and more frequent use of rescue medication, as well as a lower likelihood of receiving combined ICS + LABA therapy. This observation should be interpreted with caution, as treatment patterns may be influenced by clinical decision-making, disease management strategies, or patient adherence, rather than reflecting a direct biological effect of the variant. Additionally, age and sex related differences may also influence pharmacological response and clinical expression in severe asthma. Previous studies have shown that female patients can exhibit enhanced IL-4-related immune responses and increased type 2 inflammation, findings that have been associated with greater disease severity and increased corticosteroid requirements compared with males [28].

Notably, under the dominant model, the TG/GG genotype of rs2243248 was associated with sensitization to epithelial allergens, these findings support the notion that *IL4* SNVs act as modulators of clinical expression rather than primary determinants of disease susceptibility. A study conducted in a Pakistani population further supports this interpretation, as it reported that the T allele and the CT and TT genotypes were associated with an increased risk of asthma, and that the TT genotype was linked to significantly higher serum IL-4 levels [29]. From a biological perspective, this is consistent with the proposed regulatory role of rs2243250 and rs2243248, which may affect transcription factor binding and promoter activity. This effect may lead to increased IL-4 production, thereby amplifying Th2-mediated immune responses and contributing to clinical manifestations such as symptom exacerbation and poor disease control.

Consistent with this interpretation, no significant association was found between any haplotype combination and SA. However, evidence from other populations suggests that the combined effect of multiple regulatory variants within the *IL4* promoter region may play a more relevant role in disease susceptibility. For instance, in an Iranian population where the same variants were evaluated, the rs2070874 (−33) variant was also included in the analysis. In that study, the T allele of rs2243250, the GT genotype of rs2243248, and specific haplotype combinations were associated with asthma susceptibility. Notably, the TCT and GTC (−1098/−589/−33) haplotypes were more frequent among patients, and the TTT/GCC diplotype was associated with higher total IgE levels, suggesting a regulatory effect of the *IL4* promoter on type 2 immune responses [30]. These findings support the notion that the interaction between multiple regulatory variants may have a greater functional impact than individual SNVs alone, potentially explaining the lack of association observed in our study. Genome-wide association studies (GWAS) have shown that most risk variants are located in non-coding regions, particularly within regulatory elements [6], highlighting the importance of regulatory architecture rather than SNVs effects in complex diseases such as asthma.

In line with this, previous studies have also implicated these SNVs in related inflammatory conditions. For instance, their involvement has been explored in nonsteroidal anti-inflammatory drug–exacerbated respiratory disease (NERD), where specific genotypes have been associated with increased susceptibility to airway inflammation [23]. Furthermore, variants in *IL4* have been linked to a range of immune-mediated diseases across different populations, including allergic rhinitis, tuberculosis, atopic dermatitis, and periodontitis. However, these associations are not consistent across all populations [12,31,32,33]. As described, asthma is a heterogeneous and multifactorial disease influenced by genetic, environmental, and epigenetic factors [34]. Since *IL4* variants may contribute broadly to atopic predisposition, comparisons between patients with severe asthma and atopic individuals without asthma, such as patients with allergic rhinitis alone, could help distinguish genetic factors associated with general atopy from those more specifically related to asthma severity and clinical expression. Overall, these observations reinforce the concept that asthma is a heterogeneous and multifactorial disease influenced by genetic, environmental, and epigenetic factors [28]. Evidence from epidemiological studies highlights the contribution of environmental exposures to disease risk. For instance, exposure to biomass smoke has been reported to be more frequent among asthma patients compared to the reference group and to show a significant association with the disease [35,36]. These observations emphasize that, even within the same population, the interaction between environmental exposures and genetic predisposition can shape both disease susceptibility and severity [19].

In this context, the role of epigenetic mechanisms in asthma development has gained increasing attention. Environmental factors such as pollution, diet, allergens, and early-life infections can induce epigenetic modifications that regulate gene expression without altering the DNA sequence. These modifications represent a stable, and in some cases heritable, mechanism through which environmental signals influence immune responses. The concept of the “exposome” has further expanded this framework, encompassing the cumulative impact of environmental exposures across the lifespan and their epigenetic imprint on disease susceptibility [6]. Regulatory SNVs such as rs2243250 and rs2243248 may contribute to local regulatory environments by modifying transcription factor binding, which could in turn affect epigenetic processes such as chromatin accessibility and gene expression.

Taken together, these findings suggest that while genetic factors contribute to the development of asthma, environmental exposures may modulate its course and progression, providing a more comprehensive explanation for the variability observed across population-based studies [26,34].

The in silico analysis proved to be a valuable tool for assessing the potential functional and regulatory effects of these variants. Using bioinformatics resources such as RegulomeDB [37], and HaploReg [38], both SNVs, rs2243250 and rs2243248, were classified with a rank of 1f and a score of 0.55436, providing strong evidence of their regulatory role. According to the RegulomeDB classification system, rank 1f indicates variants likely to affect regulatory binding and linked to gene expression through evidence such as transcription factor binding, DNase peaks, and eQTL associations [34]. Additionally, lower RegulomeDB scores reflect increasing evidence that a variant is located within a functional regulatory region [37].

From a functional perspective, previous studies have suggested that the rs2243250 variant may influence transcriptional activity by modifying the binding affinity of transcription factors such as NFAT, a key regulator of IL-4 expression in activated T cells [8]. However, in the present in silico analysis, NFAT binding was not identified among the predicted interactions. Instead, regulatory evidence supported the involvement of transcription factors such as ZFX, ARID1B, and members of the Sox and MAX families.

This apparent discrepancy may reflect differences between experimentally validated mechanisms and computational predictions, as well as context-specific regulatory interactions that depend on cell type, chromatin state, or environmental stimuli. Therefore, while NFAT has been previously implicated in *IL4* regulation, our findings suggest that additional transcription factors may also contribute to the regulatory landscape of this gene.

In line with these findings, these factors, by promoting cell proliferation and survival as well as remodeling chromatin to increase DNA accessibility, may contribute to a regulatory environment compatible with increased *IL4* transcriptional activity. This regulatory context could be relevant to pathways involved in Th2 polarization and airway remodeling, both characteristic features of asthma. Additionally, these SNVs alter motifs of the Sox and MAX families. The Sox family includes several transcription factors that regulate cell differentiation and immunity, potentially modulating the Th2 response, while MAX forms dimers with Myc and other regulators, controlling genes involved in proliferation and the cell cycle [39,40]. Taken together, the presence of rs2243250 and rs2243248 may facilitate the binding of multiple transcription factors, influencing *IL4* regulatory activity in a context relevant to asthma. This supports the notion that these variants may act as regulatory enhancers of *IL4* expression under specific biological conditions, although this represents only one possible scenario and underscores the need for experimental validation. The results presented here provide an initial insight into the role of these SNVs in Mexican patients with SA.

This study provides a basis for continuing to explore the genetic predisposition underlying SA. Considering its multifactorial and polygenic nature, future research could incorporate additional molecular and clinical data, including the analysis of other genes related to Th2-driven inflammation, circulating protein levels, and environmental factors. Such integrative approaches may help clarify how *IL4* variants influence disease development. Nevertheless, these observations are based on bioinformatic predictions and should be interpreted cautiously until confirmed by functional and expression-based studies.

### Study Limitations

Nonetheless, some limitations should be acknowledged when interpreting these findings. The relatively small sample size warrants caution, as it may limit the statistical power and generalizability of the results. As shown in the sensitivity power analysis (Section 2.5), modest genetic effects (OR < 1.5), which are commonly observed for variants involved in complex diseases, may have remained undetected, which could partly explain the lack of overall association with SA susceptibility. Moreover, although the significant subgroup associations remained statistically significant after false discovery rate (FDR) correction for multiple comparisons, the relatively small sample size may still limit the precision of the estimated effect sizes; therefore, these findings should be interpreted with caution until replicated in larger and ethnically diverse cohorts. Replication in larger and ethnically diverse cohorts will be essential to confirm both the magnitude and clinical relevance of these associations [14]. Additionally, the cross-sectional design of the study precludes causal inferences between genetic variation and clinical outcomes.

The intrinsic heterogeneity of asthma, together with factors such as age, sex, comorbidities, and environmental exposures, may also have contributed to the variability observed. Moreover, since the analysis was restricted to two promoter variants of *IL4*, the potential involvement of additional genetic variants or gene and environment interactions cannot be excluded. An additional limitation is the lack of measurement of circulating IL-4 protein levels, which would have strengthened the biological interpretation of the observed genetic associations and their relationship with clinical phenotypes. Future investigations integrating larger and more diverse samples and functional genomic approaches will be essential to confirm these associations and to elucidate the molecular mechanisms underlying asthma heterogeneity.

## 4. Materials and Methods

### 4.1. Study Subjects

A total of 286 individuals over 18 years of age with Mexican mestizo ancestry were included, defined by the National Institute of Anthropology and History (INAH) as “individuals born in Mexico, of the third generation including themselves and descendants of the original native inhabitants of the region, as well as individuals primarily of Spanish descent” [41]. We conducted a cross-sectional study confirmed by 106 patients diagnosed with SA from Departamento de Inmunología Clínica y Alergia del Hospital de Especialidades, Centro Médico Nacional de Occidente, Instituto Mexicano del Seguro Social (IMSS), and 180 healthy individuals from the same geographic region were carefully selected through a structured questionnaire that included age, sex, place of residence, detailed information regarding personal history of allergic and respiratory diseases, as well as family history of asthma and atopic conditions across two generations. Individuals reporting a history of atopy, chronic respiratory disease, or a relevant familial background of these conditions were excluded from the reference group in order to minimize potential bias. However, detailed clinical evaluations such as pulmonary function tests and serum IgE measurements were not performed in individuals from the reference group. SA was diagnosed and classified according to the Global Initiative for Asthma (GINA) recommendations. Patients were considered to have SA when they required high-dose inhaled corticosteroids combined with a second controller and/or systemic corticosteroids to maintain asthma control or remained uncontrolled despite this treatment [42]. Patients were invited to participate during their clinical consultation, and after a detailed explanation of the study objectives, clinical and sociodemographic data were collected through structured questionnaires administered to both groups. The variables analyzed included age, sex, and body mass index (BMI), as well as lifestyle factors such as tobacco use. In patients with SA, clinical characteristics such as nighttime symptoms, asthma control status, intercrisis symptoms, wheezing according to level of exertion, and use of rescue medication were assessed. Additionally, comorbidities including allergic rhinitis, chronic rhinosinusitis with and without nasal polyps, aspirin-exacerbated respiratory disease, chronic obstructive pulmonary disease, diabetes mellitus, obstructive sleep apnea, and urticaria were recorded. Pharmacological treatments, including inhaled corticosteroids combined with long-acting β2-agonists (ICS–LABA), triple therapy (ICS–LABA–LAMA), oral corticosteroids, and biologic agents, were also documented. Finally, pulmonary function parameters and laboratory variables such as forced expiratory volume in one second (FEV1), forced vital capacity (FVC), the FEV1/FVC ratio, and serum immunoglobulin E (IgE) levels were included.

### 4.2. Molecular Analysis

A total of 10 mL of peripheral blood were collected in tubes containing ethylenediaminetetraacetic acid (EDTA) and genomic DNA was extracted using the modified Miller method [43]. DNA quantification was performed by spectrophotometry using a NanoDrop 2000 (Thermo Scientific, Wilmington, DE, USA). Genotyping was performed by qPCR using the allelic discrimination method with TaqMan^®^ probes (Applied Biosystems, Thermo Fisher Scientific, Waltham, MA, USA). The probe sequence for rs2243250 (C___2228458_10) was CGGATTCAGCTCCTGGATCCAGGTG[C/T]AGTGGGCTCCCAGCTTTTTCTCTCC, and for rs2243248 (C__16176227_10) it was GGGCTGATTTGTAAGTTGGTAAGAC[G/T]GTAGCTCTTTTTCCTAATTAGCTGA. Each reaction was prepared with TaqMan™ Genotyping Master Mix, Lot No. 2942011 (Applied Biosystems, Thermo Fisher Scientific, Waltham, MA, USA), according to the manufacturer’s instructions, in a final volume of 5 µL. Reactions were run on the LightCycler 96 system (Roche Diagnostics, Mannheim, Germany), and all experiments included negative controls and duplicates to ensure the reliability of genotyping.

### 4.3. In Silico Analysis

The functional analysis of the rs2243250 and rs2243248 variants was performed using RegulomeDB, (https://www.regulomedb.org/, accessed 10 February 2026). For each variant, the rank, score, experimental evidence of transcription factor binding, chromatin accessibility, and associated epigenetic states were documented. Annotation was then complemented with HaploReg v4.2, (https://pubs.broadinstitute.org/mammals/haploreg/haploreg.php, accessed 10 February 2026).

This resource allowed the identification of transcription factor binding motifs potentially altered by the variants, as well as the distribution of histone marks indicative of regulatory activity. RegulomeDB integrates data from ChIP-seq, DNase-seq, chromatin states, and eQTLs, generating a score (rank/score) that summarizes the likelihood of regulatory function. HaploReg v4.2 enables exploration of regulatory variants and their effects on transcription factor binding motifs, in addition to providing information on histone marks associated with enhancer or promoter activity.

### 4.4. Statistical Analysis

Categorical variables were presented as frequencies and percentages and analyzed using the Chi-square test or Fisher’s exact test, as appropriate. Continuous variables were expressed as means and interquartile ranges (IQR 25–75) and compared using the Mann–Whitney U test. Genotypic and allelic frequencies of the variants were obtained by direct counting, and Hardy–Weinberg equilibrium was evaluated in the reference group using the Chi-square test. Associations between genotypes and clinical characteristics were initially assessed using bivariate logistic regression models. Odds ratios (OR) and 95% confidence intervals (95% CI) were estimated under codominant, dominant, recessive, and allelic inheritance models. Additional multivariate logistic regression analyses adjusted for age, sex, and BMI were performed to further explore potential confounding effects. To account for multiple comparisons in the subgroup association analyses, false discovery rate (FDR) correction was applied using the Benjamini–Hochberg method. Adjusted *p*-values (pFDR) < 0.05 were considered statistically significant. Haplotype and linkage disequilibrium (LD) analyses were performed using the online SHEsis tool [44] (http://analysis.bio-x.cn/myAnalysis.php, accessed 10 February 2026). A sensitivity power analysis was performed to estimate the minimum detectable odds ratios according to the available sample size, minor allele frequencies, and a significance level of 0.05 under a two-sided log-additive model. Statistical power estimates were calculated for both the case–reference group comparison and subgroup analyses within the SA cohort using the pwr package (v1.3-0).

All statistical analyses were conducted in the R environment, version 4.3.1 (R Foundation for Statistical Computing, Vienna, Austria). A *p*-value < 0.05 was considered statistically significant.

### 4.5. Ethical Considerations

The study protocol was approved by the Comité de Ética del Centro Universitario de Ciencias de la Salud, Universidad de Guadalajara (approval code: CI-07024). Written informed consent was obtained from all participants prior to enrollment. All procedures were conducted in accordance with the principles of the Declaration of Helsinki, and participants received detailed written information about the objectives and scope of the study.

## 5. Conclusions

In conclusion, the present study demonstrates that the *IL4* promoter SNVs rs2243250 and rs2243248 are not associated with overall susceptibility to SA in a Mexican population, but suggest an association with specific clinical phenotypes, including nocturnal symptoms, rescue medication use, and sensitization to epithelial allergens. These findings suggest that regulatory variants in *IL4* may contribute to disease heterogeneity by modulating clinical expression rather than acting as primary genetic determinants of disease onset.

From a biological perspective, the observed associations are consistent with the proposed regulatory role of these SNVs, which may affect transcription factor binding and *IL4* gene expression, thereby enhancing Th2-mediated immune responses. This is further supported by in silico analyses indicating that these variants are located within regulatory regions with potential functional relevance.

Taken together, these results support a model in which *IL4* regulatory variation exerts context-dependent effects on asthma, shaped by the interaction between genetic background and environmental exposures. This integrative perspective highlights the importance of considering both genetic and non-genetic factors in understanding the complexity of asthma pathophysiology.

Future studies involving larger and ethnically diverse populations, as well as functional and epigenetic approaches, will be essential to validate these findings and to further elucidate the molecular mechanisms underlying asthma heterogeneity.

## Figures and Tables

**Table 1:** Sociodemographic characteristics of both study groups.

Variable	SA *n* = 106 (%)	RG *n* = 180 (%)	*p*
Age (years) ^a,b^	51 (44–60)	40 (31–50)	<0.01
Sex ^c^			<0.01
Female, *n* (%)	82 (77)	98 (54)	
Male, *n* (%)	24 (23)	82 (46)	
BMI ^a,b^	30.57 (25.75–34.23)	26.5 (24.3–29.3)	<0.01

SA: severe asthma; RG: reference group; BMI: body mass index; ^a^ Mean and interquartile range (IQR 25–75), ^b^ Mann–Whitney U test. ^c^ Chi-square test.

**Table 2:** Pharmacological treatment and comorbidities of patients with SA (n = 106).

Variable	*n*	%
Pharmacological treatment		
ICS–LABA	71	66
ICS–LABA–LAMA	28	26
Oral corticosteroids	45	42
Biologics	44	41
Comorbidities		
AERD	30	28
Allergic rhinitis	73	68
COPD	4	3
CRSwNP	48	45
DM	18	16
OSA	19	17
Urticaria	15	14
CRS	39	36

ICS: Inhaled Corticosteroids; LABA: Long-Acting β2-Agonists; LAMA: Long-Acting Muscarinic Antagonists; OSA: Obstructive Sleep Apnea Syndrome; AERD: Aspirin-Exacerbated Respiratory Disease; CRSwNP: Chronic Rhinosinusitis with Nasal Polyps; CRS: Chronic Rhinosinusitis.

**Table 3:** Clinical characteristics of patients with SA.

Variable	*n* = 106	%
Nighttime symptoms		
>2 times a week	68	64
≤2 times a week	38	36
Intercrisis symptoms		
Mild	70	66
Frequent	36	34
Wheezing		
With intense efforts	45	42
With moderate efforts	42	39
With minimal efforts	19	17
Uncontrolled asthma		
Yes	57	54
No	49	46
Relief medication		
≥3 times a week	50	47
≤3 times a week	56	53
Allergies		
Environmental inhalants	85	80
Spores	19	17
Dandruff and epithelia	11	10
Food	10	9
Pharmacological	8	7
Variable	Mean	
FEV1	70.31 (55.25–83.75)	
FVC	89.56 (71–96.75)	
FEV1/FVC	64.97 (59.09–74.17)	
IgE (IU/mL)	293.72 (38.5–346)	

Mean and interquartile range (IQR 25–75). FEV1: forced expiratory volume in the first second; FVC: forced vital capacity; FEV1/FVC: ratio of forced expiratory volume in the first second to forced vital capacity, IU/mL: International Units per milliliter.

**Table 4:** Genotypic and allelic frequencies of rs2243250 and rs2243248 variants of *IL4* in the studied groups.

Variant			SA		RG		OR	95% CI	*p*
	Model	Genotype	*n* = 104	%	*n* = 178	%			
rs2243250	Codominant	CC	32	30	44	25	1	—	—
		CT	55	53	93	52	0.81	0.46–1.43	0.47
		TT	17	17	41	23	0.57	0.28–1.18	0.15
	Dominant	CC	32	30	44	28	1	—	—
		CT + TT	72	70	134	72	0.74	0.43–1.27	0.27
	Recessive	TT	17	16	41	23	0.65	0.35–1.22	0.22
		CC + CT	87	84	137	77	1	—	—
	Alleles	C	119	57	181	51	1	—	—
		T	89	43	175	49	0.77	0.54–1.11	0.16
rs2243248	Codominant	TT	83	80	142	80	1	—	—
		TG	21	20	35	19	1.03	0.53–1.95	1
		GG *	0	0	1	1	—	—	—
	Dominant	TT	83	79	142	79	1	—	—
		TG + GG	21	21	36	21	1	0.52–1.89	0.99
	Recessive	GG *	0	0	1	1	—	—	—
		TT + TG	104	100	177	99	1	—	—
	Alleles	T	187	90	319	89	1	—	—
		G	21	10	37	11	0.97	0.55–1.70	0.99

OR: odds ratio, CI: 95% confidence interval, significance: *p* < 0.05, * Estimates were not calculated due to low or zero cell counts.

**Table 5:** Associations between *IL4* variants and clinicopathological variables in the SA group.

Variant	Genotype Comparison	Variable	OR	95% CI	*p*	*p*FDR
rs2243250	TT vs. CC + CT	Nighttime symptoms	3.03	1.31–7.00	0.0096	0.020
rs2243250	TT vs. CC + CT	Rescue medication	3.16	1.41–7.07	0.0052	0.019
rs2243250	TT vs. CC + CT	ICS + LABA (Budesonide)	0.15	0.03–0.76	0.023	0.030
rs2243248	TG + GG vs. TT	Epithelial allergy	3.96	1.07–14.6	0.039	0.039

Bivariate logistic regression analysis. OR: odds ratio, CI: 95% confidence interval, significance: *p* < 0.05, *p*FDR: false discovery rate-adjusted *p*-value.

**Table 6:** Haplotype frequency of the groups.

Haplotype	SA	RG	OR (95% CI)	*p*
C G	0.101	0.098	1.02 (0.58–1.81)	0.9417
C T	0.471	0.410	1.27 (0.90–1.79)	0.1730
T G	0.000	0.005	–	–
T T	0.428	0.486	0.78 (0.55–1.10)	0.1621

OR: odds ratio, CI: 95% confidence interval.

**Table 7:** Regulatory evidence for rs2243250 and rs2243248 in *IL4* according to RegulomeDB and HaploReg.

Variant	Rank/Score	TF Binding (ChIP)	Altered Motifs (HaploReg)	Chromatin State (Summary)	Accessibility (Examples)	Interpretation
rs2243250	1f/0.55436	ZFX, ARID1B	Sox, MAX	Active enhancer	Immune/vascular tissues and T47D cell line; accessibility in multiple tissues	Consistent regulatory evidence
rs2243248	1f/0.55436	ZFX, IKZF1	Osr	Active enhancer	Frontal cortex, coronary artery, activated CD8+ T cells, lung	Consistent regulatory evidence

## Data Availability

The original contributions presented in this study are included in the article. Further inquiries can be directed to the corresponding author.
